# Foodborne Illness Outbreak Investigation in a High-Profile Sports Club

**DOI:** 10.1186/s40798-017-0088-x

**Published:** 2017-06-24

**Authors:** Kwendy Cavanagh, Travers Johnstone, Essi Huhtinen, Zeina Najjar, Peter Lorentzos, Craig Shadbolt, John Shields, Leena Gupta

**Affiliations:** 1 Sydney Local Health District Public Health Unit, King George V Building, Missenden Road, Camperdown, NSW 2050 Australia; 2 New South Wales (NSW) Food Authority, 6 Avenue of the Americas, Newington, NSW 2127 Australia; 3 Sports team physician, Sydney, Australia

**Keywords:** Public health, Communicable diseases, Sports, Disease outbreaks, Athletes, Salmonella, Foodborne illness

## Abstract

A foodborne illness outbreak involving an elite sports team was investigated by a public health unit in Sydney, Australia. An epidemiological association was established between gastrointestinal illness and the consumption of food supplied by an external caterer, with a lamb meal most strongly associated with illness. Genetically identical *Salmonella* isolates were identified from clinical specimens, residual food items, and an environmental swab taken from the catering premises. The training schedule and other club operations were significantly affected by this outbreak. Increased susceptibility due to regular shared activities and the potential for significant impact upon performance indicates that sports clubs must ensure that food suppliers comply with the highest standards of hygiene. Collaboration with public health authorities assists in source identification and prevention of further transmission.

## Key Points

- The impact of foodborne illness on sports teams can be significant, and foodborne illness is likely to be underreported amongst athletes.
- Sports teams requiring catering for elite athletes and support staff should ensure that food suppliers follow strict food safety procedures and should consider obtaining written evidence of adherence.
- A prompt notification to relevant health authorities enabling a timely investigation will limit risk to the team and the wider community.

## Background

An estimated 4.1 million cases of foodborne illness are reported in Australia each year, and outbreaks are common [1]. Despite the frequency of such outbreaks in the general population [1, 2], infectious disease outbreaks of any type in sporting teams are not frequently described in the literature [3–5]. The few reported outbreaks in sporting teams in Australia have mainly been caused by environmental pathogens, such as *Cryptosporidium* [6, 7] and *Aeromonas* hydrophila [8], rather than foodborne organisms. In Australia, only two published outbreaks relating to food and sports were identified on review; an outbreak of gastroenteritis amongst six persons associated with a team lunch [9] and a *Staphylococcus aureus* outbreak amongst 22 participants of an elite event [10]. Internationally, reports of gastroenteritis in sports teams have been linked to ice used in drinks [11], a football game [12], a rowing tournament [13] and more recently, an outbreak of *Salmonella* affecting a number of European ice hockey teams was linked to catering venues at the event [13]. However, such accounts are not numerous, suggesting that foodborne illness outbreaks in sporting teams are likely to be significantly underreported.

Sports teams may be at increased risk of infectious diseases due to their close physical interactions, exposing them to both point-source and person-to-person transmission [3, 4, 12]. Teams may also be at risk of foodborne illness because of mass catering at training, events or functions. Furthermore, when food is prepared for specific dietary circumstances or needs of sportspersons, adherence to strict food safety principles is required. Large-scale sporting events will often have risk management plans which include food safety as a significant area for risk mitigation [14]. However, when catering is on a smaller, more routine scale, such plans or consideration of food safety may not be a particular priority.

This report describes an outbreak of *Salmonella* amongst the team and staff of a professional sports club during training season, the challenges involved in the investigation, and practical implications for future prevention and management of similar incidents amongst sportspersons.

## Case Report

In late 2014, a public health unit (PHU) in Sydney was notified by the doctor of a professional sports team of 33 players and staff at a sports club who had developed gastrointestinal symptoms within a 24-h period. In New South Wales (NSW), Australia, suspected foodborne illness outbreaks are notifiable to public health authorities under the *NSW Public Health Act 2010* [15]. In line with NSW health guidelines [16], an outbreak investigation was initiated in conjunction with the NSW Food Authority (NSW FA), the lead food regulatory agency, to determine the cause and control any ongoing risk.

**Fig. 1:**
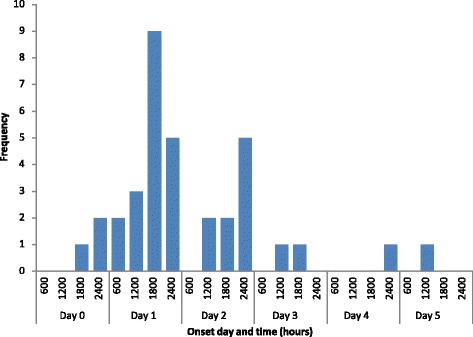
Epidemiological curve showing onset of gastrointestinal illness in members of the sports club (*n* = 35)

The PHU conducted a retrospective cohort study, with the cohort being defined as all individuals who attended the sports club premises within a 72-h period surrounding the first reported onset of illness. A case was defined as a member of the cohort who developed gastrointestinal symptoms within the 7 days following their attendance at the club. A modified standard questionnaire [17] was used to collect details of any illness and of foods consumed. Ninety-one people attended the club during this period, forming the cohort; 76 of these were interviewed (response rate 84%). Of those interviewed, 35 people were identified as cases, of which 17 (49%) were players and 18 (51%) were staff. Frequencies were calculated for symptoms, with the most common being diarrhoea (97%), abdominal cramps (83%) and fever (74%). Distribution of symptom onsets are demonstrated in Fig. 1: Epidemiological curve showing onset of gastrointestinal illness in members of the sports club (*n* = 35).

Each day, a catering company supplied one type of meal and a supplementary snack to the club. These meals were generally reheated by microwave at the club, though some were consumed cold following nutritional advice. Relative risks (RR) and 95% confidence intervals (CI) were calculated for each meal item, as shown in Table 1.

**Table 1:** Catered meals consumed by sports club attendees

Meals consumed (day)	Total club attendees	Total attendees consuming meal	Unwell (%)	Well (%)	Relative risk* (95% C.I.)
Chicken meal (Day–1)	72	34	22 (65%)	12 (35%)	2.09 (1.25, 3.50)
Lamb meal (Day 0)	67	35	31 (89%)	4 (11%)	9.08 (3.55, 23.21)
Pork meal (Day + 1)	45	8	7 (88%)	1 (12%)	2.33 (1.55, 3.51)
Supplementary snack (daily)	N/A	28	20 (71%)	8 (29%)	2.19 (1.36, 3.53)

*Reference group is club attendees on the same day that did not eat the specified meal

Consumption of the lamb meal had the strongest significant association with illness, with the other meals also significantly associated with illness, though less strongly. Additionally, the club reported that due to illness, attendance at training dropped to approximately 70% in the week post-outbreak, and full attendance was not regained until 2 weeks post-outbreak.

An environmental investigation was undertaken by NSW FA. This involved site visits to the sports club and to the catering premises that provided ready-to-eat meals to the club; environmental and food samples were collected from both premises. The inspection of the caterer found that the business had adequate processes for cooking of foods, and the cleaning and sanitising of the premises was of a satisfactory standard. Although disposable gloves were changed regularly, staff reported that this was not always in conjunction with regular hand-washing. An available chicken meal was reheated in the club microwave to test the meal label instructions; the chicken component of the meal was found to be at an inadequate temperature of between 28 and 42 °C (higher than 60 °C is recommended) [18].

All cases were encouraged to submit a stool sample for microbiological testing. Faecal, food and environmental samples underwent microbial analysis. Serotyping and genetic sequencing, in the form of multiple-locus-variable number tandem repeats analysis (MLVA) [19, 20] were performed on all clinical, food and environmental isolates positive for *Salmonella* species at a reference laboratory. The day after the outbreak was reported, the first stool sample returned positive for *Salmonella* and over the following week, the remaining nine stool samples also tested positive for *Salmonella*. These were later all typed as *Salmonella enterica* serovar *typhimurium*, MLVA 3-12-11-14-523. Four residual meals (two lamb, two pork) from the club premises tested positive for *S. typhimurium*, two with an identical MLVA to the clinical specimens. Quality control and constituent component food samples from the caterer were tested and found to be satisfactory. One environmental swab was found positive for *S. typhimurium,* again with an identical MLVA to the matching clinical and food isolates.

## Discussion

This investigation identified a significant point-source foodborne outbreak of *S. typhimurium* amongst members of an elite sports team and the club’s staff, with a strong epidemiological association between gastrointestinal illness and the consumption of food prepared by an external catering company. The evidence of an epidemiological link is supported by microbiological evidence of genetically identical isolates from clinical samples, one residual lamb meal, a pork meal and an environmental swab from the catering company premises. Thirty-one team members and staff had symptom onsets ranging from 6 to 58 h after consumption of the lamb meal, consistent with the usual incubation period for *Salmonella* [21].

It was postulated that the outbreak occurred due to contamination of individual food portions during preparation of batches made specifically for the sports club, as there were no other reports of illness related to this caterer during this time period. Furthermore, whilst reheating the meals would have afforded some protection, many members ate the meals cold and the reheating procedures tested at the club did not heat the food to sufficient temperatures to render it safe [18].

The impact of a foodborne illness on athletes has the potential to disrupt training and competition schedules. The attack rate in this outbreak was 38% amongst club members, slightly lower than a previously reported outbreak of *Salmonella* at a world rowing tournament which affected up to 60% of some teams, preventing participation of those affected [22]. It was fortunate that this outbreak occurred during the pre-season training period, which allowed players more flexibility to isolate themselves and rest adequately. However, the pre-season is also a period dedicated to building strength and weight in preparation for the coming season. In the opinion of the coaching staff of the sporting club, the outbreak had an impact on the team’s long-term performance during the following season, due to weight loss amongst some players.

Fortunately, this outbreak was identified and notified by the team physician so that public health action could be taken to prevent further transmission and avert future outbreaks from this particular source. However, outbreaks of this nature can be underreported for a number of reasons, including lack of knowledge of reporting requirements or use of different healthcare providers by different members of sports teams. Notification of illness in elite athletes may also be hindered by concerns regarding confidentiality [23] and potential media exposure; noting that the media has previously reported upon foodborne illness in high-profile teams [24, 25]. Furthermore, public health investigations amongst sportspersons can be challenging because team doctors or the sportspersons themselves may be reluctant to divulge necessary identifying or clinical information because of the public profile of the team or individuals.

There were a number of limitations to our study. As most cases were interviewed whilst they were still unwell, it was not possible to ascertain the length of illness and hence determine the overall impact of the outbreak on the team and its training goals. In addition, obtaining the sensitive, yet critical, clinical information was a challenge for the investigation team. There were delays in obtaining contact information for some individuals who had attended the club but were unaffected by illness. Although the first interviews were conducted the day following notification to the PHU, the final interviews were not completed until 18 days later. This may have affected the interviewee’s ability to recall their meals and activities during the period of interest, potentially introducing recall bias. However, this may have been mitigated by the use of a standard questionnaire.

This outbreak highlights the importance of considering food safety when organising mass catering for elite athletes, where the physical health of participants is paramount. Although cross-contamination incidents can occur randomly, clubs can reduce the risk of foodborne illness outbreaks by ensuring that external caterers have appropriate food safety procedures in place. This could include requesting evidence of food safety training or inspection reports. The hazard analysis and critical control point (HACCP) risk management system [26], designed to control food safety hazards, is used across a wide range of settings, including at large sporting events [14]. Organisations should consider using HACCP-accredited companies when selecting food suppliers for mass catering.

## Conclusions

This point-source outbreak of *S. typhimurium* impacted considerably upon the operations of a professional sports club. The frequent communal activities undertaken in sports clubs, coupled with the requirements for peak physical health, may cause them to be significantly affected by an outbreak of foodborne illness, despite the general good health of their members. In the first instance, stringent investigation of prospective food suppliers is recommended and if incidents do occur, prompt reporting to public health authorities will assist in halting further transmission.

## Abbreviations

HACCP: Hazard analysis and critical control points
MLVA: Multi-locus-variable number tandem repeats analysis
NSW: New South Wales
NSW FA: New South Wales Food Authority
PHU: Public health unit
